# In the U.S. “Healthcare” Is Now Strictly a Business Term

**DOI:** 10.5811/westjem.2018.1.37540

**Published:** 2018-03-13

**Authors:** Nick T. Sawyer

**Affiliations:** University of California, Davis, Department of Emergency Medicine, Sacramento, California, California American College of Emergency Physicians Board of Directors

## November 10, 2017

In the U.S., healthcare is now strictly a business term. Healthcare organizes doctors and patients into a system where that relationship can be financially exploited and as much money extracted as often as possible by hospitals, clinics, health insurers, the pharmaceutical industry, and medical device manufacturers. If possible, the more that patients resent their doctors, the better it is for the business of healthcare. As long as that dynamic exists, patients and doctors will never align: this would be the ultimate threat to the business of healthcare.

This adversarial patient-doctor relationship is maintained by overworking and under-supporting doctors both with regard to heaping administrative burden/caseloads on them and limiting how much help they can actually offer patients. The patients then encounter a vast number of burned-out doctors whose shining idealism once held in medical school has been slowly drawn out of them.

In primary care, patients feel they are left unheard because doctors spend just 15 minutes with them. Doctors feel as if they don’t have time to listen because they only have 15 minutes with their patients. The situation is exacerbated by TV ads that tell doctors and patients the newest pill will fix the patient’s problem: the healthcare business only needs the doctor and patient to interact just long enough for the doctor to be the conduit whereby that pill gets prescribed.

We in the emergency department proudly serve as the safety net for patients in need. We see anybody, anywhere, anytime. Along with our colleagues – the hospitalists and on-call specialists – we work tirelessly day and night to help patients. After these interactions, however, the healthcare business offers little by way of support for the patient or the doctor. And then the medical billing mechanism begins to churn. Money is requested by the hospital/clinic billing department on behalf of the doctor from the patient’s insurance or the patient directly. This process is so opaque that neither patients nor doctors can understand it, and no one will willingly explain it.

This confusion is created deliberately to obfuscate the way that hospitals, clinics, health insurers, and drug and medical device manufacturers have made billions in the business of healthcare. Publicly traded, for-profit health insurers, for example, make billions per year. As these companies’ shares are publicly traded on the New York Stock Exchange, they have a fiduciary (legal) responsibility to make money for their shareholders, not to do what’s best for the patient. That seems antithetical to any healthcare system.

Hospitals also siphon billions of dollars from the system, shifting dollars to shareholders to build new hospitals or expand capacity to increase their market share. Meanwhile, patients are sent obscene bills and blame the doctors. It’s a beautifully orchestrated scheme in which the U.S. spends more and more on healthcare – more in fact than any other country in the world – and ironically those who gain the least are the patients and the doctors.

## What can we do?

I think the first step is awareness that this is happening and getting worse. Awareness is particularly important among medical students and residents. Medical education has long ignored the business of medicine as part of undergraduate/graduate medical education, but that is starting to change. Many medical schools have started rolling out a new curriculum termed Health System Science, which is considered the “third science” along with basic sciences and clinical medicine. A recently released textbook is a good read for medical students, residents and attendings alike ( https://www.amazon.com/Health-Systems-Science-Susan-Skochelak/dp/0323461166).

I would also suggest reaching out to the American Medical Association (AMA), your AMA state chapter, or your specialty practice group (American College of Emergency Physicians, for me). Get involved at the state or national level to develop an understanding of the landscape in order to best navigate a way forward. Next, consider getting an MBA. For medical students if your medical school offers a combined degree, do it. Alternatively, consider taking time off during med school to get an MBA or pursue an MBA after residency. An advanced business degree will provide a level of understanding needed to navigate the financial chaos.

Speak up. Call or write your local congressional Representative and U.S. Senators to voice your concerns. The phone number to the U.S. Capitol switchboard is (202) 224-3121. You can find information on who represents you at this site: https://www.congress.gov/contact-us

Finally, we need to form a coalition of physicians and patients who can advocate for changes that serve our interests. I’m unaware of a strong patient-physician advocacy group at this time that has enough power to oppose the lobbying efforts of the industries noted above. It would be an important next step.

All that being said, I do not mean to equate the business of healthcare with the practice of medicine. To those of us in practice, medicine, both the science and the art, brings us great joy and purpose. We have dedicated our lives to helping others and we are nothing if not resilient. As we move forward, I do not see a simple solution to this problem, nor do I believe there is a particular set of tactics we should pursue that will help us fix this. What I will say is this - as physicians we are the true medical experts and we should not be afraid to speak up on behalf of our patients and ourselves whenever we encounter situations where the business of healthcare is placed above/or is in conflict with the practice of medicine.

